# Clinical Trial of Open-Label Mezagitamab in IgAN: Final 96-Week Results

**DOI:** 10.1016/j.ekir.2026.107128

**Published:** 2026-10-07

**Authors:** Jonathan Barratt, Dong Ki Kim, Inwhee Park, Jessica Ryan, Yosuke Saka, Mamiko Shimamoto, Yusuke Suzuki, Iwona Dobler, MK Farmer, Cheryl Li, Van Anh Nguyen, Parth Patwari

**Affiliations:** 1 Mayer IgA Nephropathy Laboratories, Department of Cardiovascular Sciences, University of Leicester, Leicester, UK; 2 John Walls Renal Unit, University Hospitals of Leicester NHS Trust, Leicester, UK; 3 Department of Internal Medicine, Seoul National University College of Medicine, Seoul, Republic of Korea; 4 Department of Nephrology, School of Medicine, Ajou University, Suwon, Republic of Korea; 5 Department of Nephrology, Monash Medical Centre, Melbourne, Victoria, Australia; 6 Department of Medicine, Monash University, Melbourne, Victoria, Australia; 7 Department of Nephrology, Kasugai Municipal Hospital, Aichi, Japan; 8 Department of Nephrology, Sapporo City General Hospital, Hokkaido, Japan; 9 Department of Nephrology, Juntendo University Faculty of Medicine, Tokyo, Japan; 10 Takeda Development Center Americas, Inc., Cambridge, Massachusetts, USA

**Keywords:** CD38, clinical study, IgA nephropathy, mezagitamab, subcutaneous, TAK-079

## Abstract

**Introduction:**

Therapeutic options that allow a substantial time off-treatment and prevent progression to kidney failure in patients with IgA nephropathy (IgAN) are limited. Mezagitamab, an anti-cluster of differentiation (CD) 38 monoclonal antibody, may deplete plasma cells that produce galactose-deficient IgA1 (Gd-IgA1), thereby stabilizing kidney function.

**Methods:**

This multicenter, open-label, single-arm, phase-1b study evaluated mezagitamab in combination with stable background therapy. Eligible participants had biopsy-proven disease, proteinuria, and estimated glomerular filtration rate (eGFR) ≥ 45 ml/min per 1.73 m^2^. Participants received subcutaneous mezagitamab 600 mg once weekly for 8 weeks, then 600 mg every 2 weeks for 16 weeks. The primary end point was the percentage of participants with adverse events (AEs) through week 96.

**Results:**

Seventeen participants enrolled. There were no serious AEs, study discontinuations because of AEs, grade ≥ 3 infections, or opportunistic infections. Rapid reductions were observed in serum IgA, IgG, IgM, and Gd-IgA1, with a sustained depletion in Gd-IgA1, but partial or complete recovery in IgA, IgG, and IgM by week 96. Proteinuria rapidly decreased within weeks, and at week 96 the urine protein-to-creatinine ratio (UPCR) was reduced by a model-based mean change from baseline of −53.4% (95% confidence interval [CI]: −72.6 to −20.7, *n* = 13). Hematuria resolved in 60% of participants with baseline hematuria. Renal function remained stable at week 96 (model-based mean change from baseline in eGFR: +3.2 [95% CI: −1.1 to +7.5; *n* = 13]).

**Conclusion:**

Mezagitamab was generally well tolerated in patients with IgAN. Stable kidney function was observed through week 96, with reductions in proteinuria, hematuria, and Gd-IgA1, approximately 1.5 years after the last dose of mezagitamab.

## Graphical abstract

**Figure:**
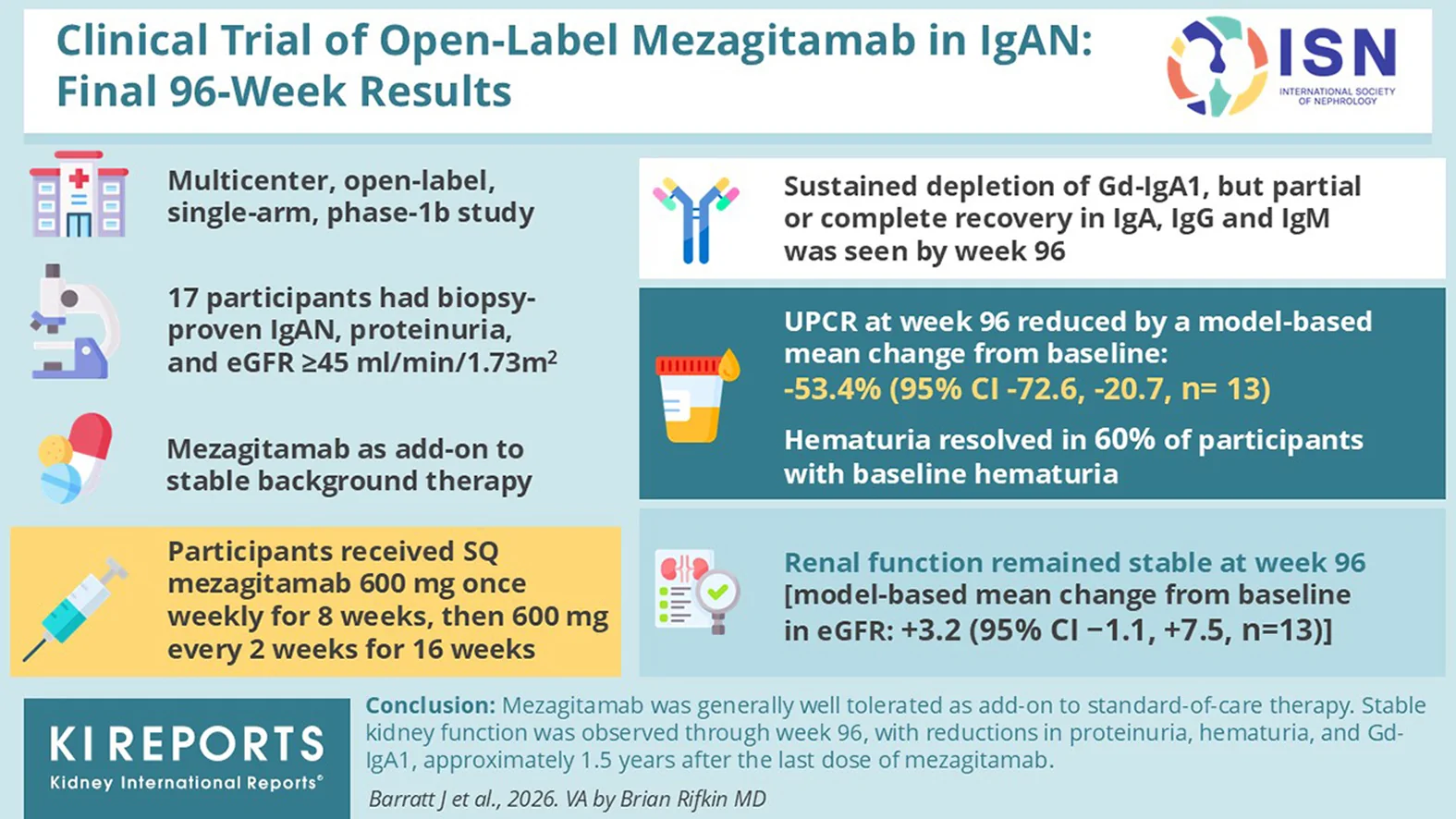

IgAN is the most prevalent form of primary glomerulonephritis worldwide.^1^, ^2^ IgAN is associated with a poor prognosis and often can lead to reduced quality of life, progress to kidney failure, or cause premature death.^3^, ^4^ Guideline-recommended therapies for IgAN have historically focused on supportive care with renin-angiotensin system inhibitors.^5^ Despite recent approvals of drugs for IgAN, there remains an unmet need for tolerable medications with disease-modifying effects that are sustained off-therapy.

Mezagitamab (TAK-079) is a fully human, anti-CD38 IgG1 monoclonal antibody. Unlike B cell–directed anti-CD20 antibodies such as rituximab, mezagitamab is expected to deplete plasmablasts and plasma cells that express high levels of CD38, while having little effect on overall B cell counts.^6^, ^7^ Mezagitamab also depletes other CD38-expressing immune cell subsets such as natural killer cells. By depleting short-lived and long-lived antibody-secreting cells, mezagitamab can produce sustained reductions in Igs of all classes, but interestingly it reduces IgA more strongly than IgG or IgM. We therefore hypothesized that mezagitamab could have strong efficacy in IgAN by acting at multiple points in the disease pathogenesis, by depleting cells responsible for producing both the pathogenic Gd-IgA1 and the antiglycan antibodies against Gd-IgA1.^6^, ^8^ Depletion of these cells may decrease formation and deposition in the kidney of IgA-containing immune complexes, potentially reducing proteinuria and promoting the stabilization of kidney function over time.

Although anti-CD38 therapy is approved for use in patients with multiple myeloma,^9^ its efficacy in nonmalignant, antibody-mediated disorders is not established. Here, we report results from the final analysis of a phase 1b study evaluating the safety, tolerability, pharmacokinetics (PK), and efficacy of subcutaneous mezagitamab in combination with stable background therapy in participants with primary IgAN. Participants were evaluated for up to 96 weeks to assess the long-term safety and durability of response.

## Methods

### Study Design

This was a multicenter, open-label, single-arm, phase 1b study (NCT05174221) in participants with primary IgAN who were receiving stable supportive care with approved renin-angiotensin-aldosterone system–blocking agents, but who still exhibited significant proteinuria. The main study consisted of a ≤ 28-day screening period, followed by a 14-day predosing period for determination of baseline proteinuria, a 24-week dosing period, and a 24-week safety follow-up (Figure 1). During the dosing period, participants received subcutaneous mezagitamab 600 mg once weekly for 8 weeks, then 600 mg every 2 weeks for 16 weeks (for a total of 16 doses). Medications including a corticosteroid, antipyretic, antihistamines, and montelukast were administered before the first dose of mezagitamab to reduce the risk of hypersensitivity reactions and injection-related reactions.

**Figure 1:**
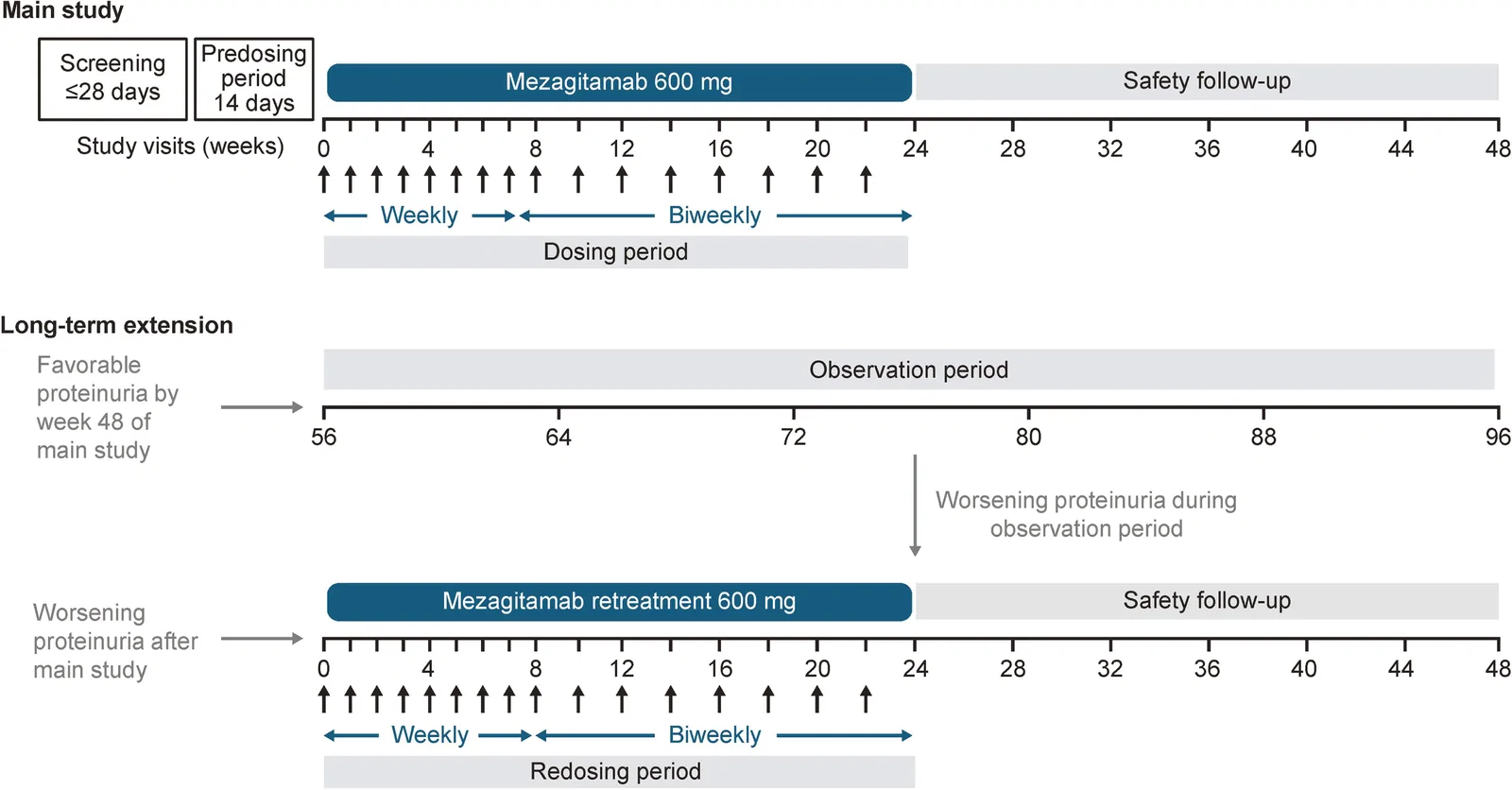
Study design.

Participants with a favorable proteinuria response by week 48 were eligible to enter the long-term follow-up, consisting of a 48-week observation period. A favorable proteinuric response was defined as follows: (1) 24-hour UPCR reduced by ≥ 10% from baseline on 2 consecutive measurements or visits; or (2) UPCR reduced by ≥ 10% from baseline on ≥ 1 24-hour urine measurement and determined by the study investigator to have an adequate proteinuric response with approval by both the investigator and study sponsor.

Participants were considered for retreatment if they had worsening proteinuria at any time after the completion of the main study, defined as follows: (1) 24-hr UPCR greater than the mean value between the baseline and the lowest 24-hr UPCR from the main study or a doubling of the lowest 24-hr UPCR from the main study (whichever was lower) and (2) 24-hr UPCR ≥ 1 g/g (or ≥ baseline if the baseline 24-hr UPCR was < 1 g/g). The retreatment period consisted of a 24-week redosing period with administration of the same regimen as in the main study, followed by a 24-week safety follow-up period.

The study was conducted in accordance with the protocol, the ethical principles of the Declaration of Helsinki, the International Conference on Harmonization Good Clinical Practice Consolidated guideline, and all applicable local regulations. All participants provided written informed consent.

### Study Population

Eligible participants were ≥ 18 years old with biopsy-proven IgAN within the last 10 years, and with proteinuria (UPCR ≥ 1 g/g from a 24-hour urine collection or urine protein excretion ≥ 1 g/24 h), stable background therapy (angiotensin converting enzymes inhibitors and/or angiotensin receptor blocker) for ≥ 12 weeks, blood pressure controlled to ≤ 140/90 mm Hg with stable antihypertensive therapy, and eGFR ≥ 45 ml/min per 1.73 m^2^ (using the Chronic Kidney Disease Epidemiology Collaboration formula 2021). Participants were excluded if they had concomitant kidney disease on biopsy; secondary IgAN; rapidly progressive glomerulonephritis; nephrotic syndrome; active extrarenal IgA vasculitis; kidney or other organ transplantation (before or expected during the study); immunosuppressant or B-cell–directed biologic therapies use within 6 months or systemic corticosteroid use within 4 months of screening or expected use during the trial; or inadequate organ and bone marrow function at screening visit (alanine aminotransferase or aspartate aminotransferase > 3 times the upper limit of normal, total bilirubin > 1.5 times the upper limit of normal, platelets < 75,000/mm^3^, neutrophil count < 1500/mm^3^, lymphocyte count < 500/mm^3^, hemoglobin < 8 g/dl, and IgG < 6 g/l).

### End Points

The primary end point was the proportion of participants with AEs, including grade ≥ 3 AEs and serious AEs up to week 96. Secondary end points included serum IgA levels and serum mezagitamab concentrations (PK) through week 96, antidrug antibody assessments, and the change from baseline to week 96 in UPCR. Exploratory end points included the change from baseline in eGFR at week 96, hematuria at prespecified time points up to week 96, serum IgG, Gd-IgA1, and IgM levels up to week 96, and change from baseline in immune cell subsets. Resolution of hematuria was defined as participants with hematuria at baseline (non-negative and/or trace assessed by point-of-care dipstick urinalysis or complete urinalysis with reflex microscopy or flow cytometry) who converted to negative and/or trace hematuria after treatment. Serum Gd-IgA1 levels were assessed at a central laboratory using a solid-phase, sandwich enzyme-linked immunosorbent assay (Gd-IgA1 Assay Kit, Immuno-Biological Laboratories Co., Ltd., Minneapolis, MN).

### Statistical Analysis

This was an exploratory study and was not powered to formally test any statistical hypothesis. Safety, efficacy, PK, pharmacodynamic, and immunogenicity analyses were performed using data from all participants who received ≥ 1 dose of study drug. End points are reported for the main study and long-term extension (LTE).

Safety end points were summarized by the number and percentage of participants with AEs and exposure-adjusted incidence rates. Efficacy, PK, pharmacodynamic, and immunogenicity end points were summarized by descriptive statistics. The change in UPCR from baseline was analyzed using a mixed model for repeated measures. The response variable was the difference between the log-transformed UPCR values post baseline and the log-transformed UPCR baseline values. Visit was included as a categorical variable and log-transformed baseline values and the interaction between baseline and visit were included as covariates. The model-based, log-transformed mean estimates and 95% CIs by visit were back-transformed using the exponential function and presented as the geometric mean and percent change from baseline with 95% CIs. Absolute change from baseline in eGFR was analyzed using a mixed model for repeated measures that included the change from baseline at each visit as the response variable, visit as a categorical variable, and the baseline values as a covariate. An unstructured covariance structure was used in the UPCR model whereas the first-order autoregressive covariance structure was used for the eGFR model because it did not converge with an unstructured covariance structure. Model-based mean estimates and 95% CIs of the eGFR change from baseline over time, up to week 96, are reported. The change from baseline in the urine albumin-creatinine ratio was analyzed in a manner analogous to the UPCR analysis. Resolution of hematuria at each postbaseline visit is descriptively summarized for the participants who had hematuria at baseline and nonmissing hematuria data for the corresponding visit.

## Results

### Participant Population

Thirty-eight participants were screened (Figure 2). Of those who were screened, 17 participants were dosed and all 17 completed the main study. After completing the main study, 3 participants did not enter the LTE—2 because of lack of proteinuric response and 1 participant for reasons related to safety (dose holds because of concern for hypersensitivity and ongoing high IgE levels). Thirteen participants continued in the LTE observation period without retreatment, whereas 1 qualified for retreatment based on the week-48 data and was dosed in the LTE retreatment period. All participants who met the LTE entry criteria qualified under criteria 1 (24-hour UPCR reduced by ≥ 10% from baseline on 2 consecutive measurements or visits). One participant in the LTE observation period later entered the retreatment period at week 96. Baseline demographics and disease characteristics are presented in Table 1. Briefly, participants had a mean (SD) age of 40.8 (13.6) years and 70.6% of them were Asian. Participants were on average 2.7 years since their most recent diagnostic kidney biopsy; histological characteristics are reported in Supplementary Table S1. At study start, 16 of 17 (94.1%) participants received an angiotensin receptor blocker or angiotensin converting enzyme inhibitor as background therapy; 35.3% of participants also received an sodium-glucose cotransporter-2 inhibitors inhibitor.

**Figure 2:**
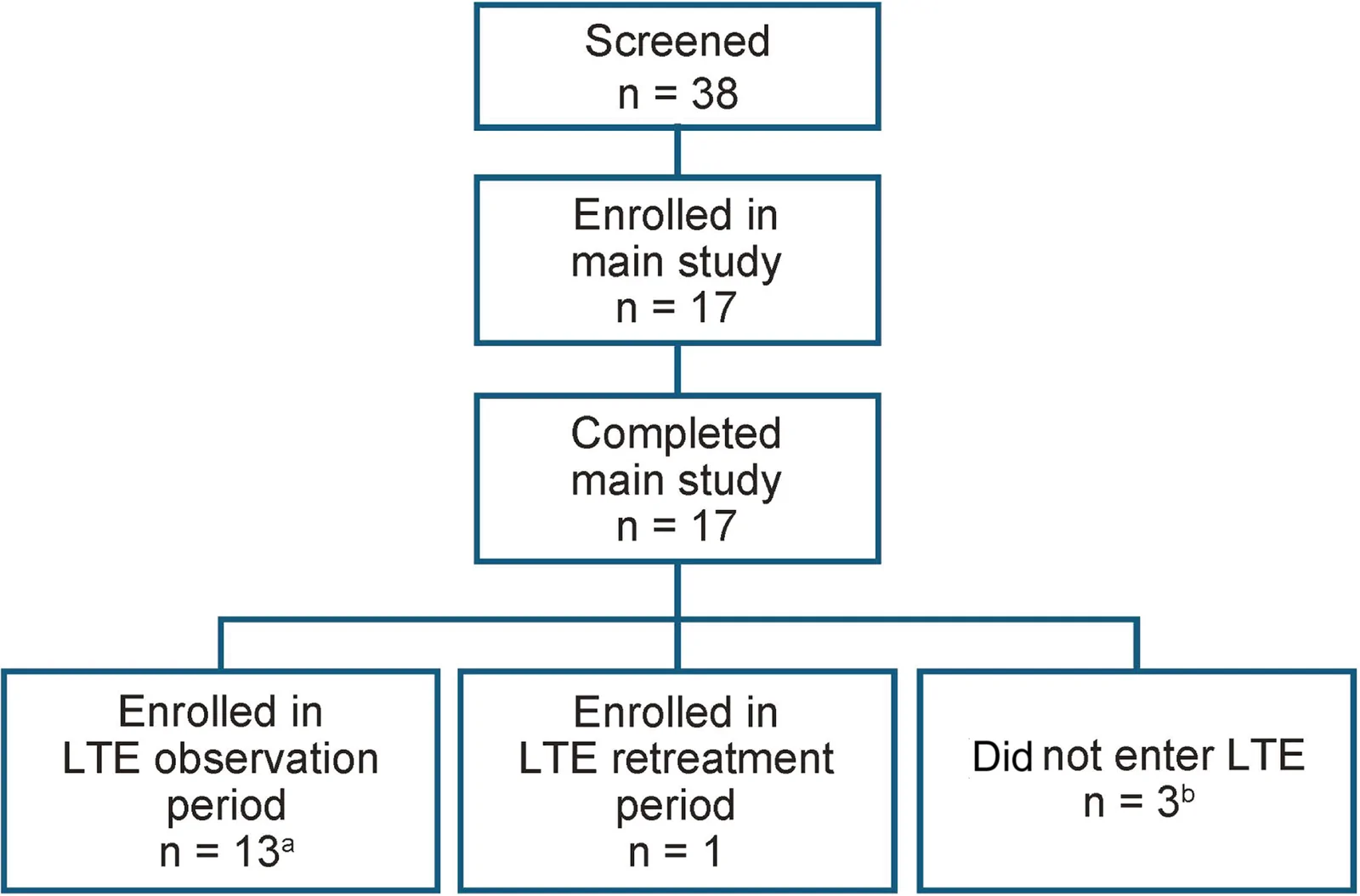
Participant flow diagram. Thirty-eight patients provided informed consent for the study and 17 were dosed. All 17 participants completed the main study. Thirteen participants subsequently entered the LTE observation period. ^a^One participant enrolled in the LTE retreatment period at week 96. ^b^Reasons for not entering the LTE—safety, lack of efficacy, patient decision. LTE, long-term extension.

**Table 1:** Baseline demographics and clinical characteristics

	Total (*N* = 17)
Demographics	
Age, yr	
Mean (SD)	40.8 (13.6)
Range	20–68
Sex, *n* (%)	
Male	8 (47.1)
Female	9 (52.9)
Race, *n* (%)	
Asian	12 (70.6)
White	5 (29.4)
BMI, kg/m^2^	
Mean (SD)	27.1 (4.2)
Range	19.1–33.0
Systolic blood pressure, mm Hg	
Mean (SD)	119.1 (12.6)
Range	104–141
Diastolic blood pressure, mm Hg	
Mean (SD)	76.0 (12.7)
Range	55–93
Background therapy, *n* (%)	
ACE inhibitor	4 (23.5)
ARB	12 (70.6)
SGLT2 inhibitor	6 (35.3)
Time since most recent biopsy, yr	
Mean (SD)	2.7 (2.9)
Range	0.2–9.4
Years since IgAN diagnosis	
Mean (SD)	4.6 (7.4)
Range	0.1–28.6
Previous tonsillectomy, *n* (%)	0
Baseline characteristics	
24-h UPCR, g/g	
Mean (SD)	1.5 (0.6)
Range	0.9–3.0
24-h UPE, g/d	
Mean (SD)	2.0 (0.9)
Range	0.7–4.1
24-h UACR, g/g	
Mean (SD)	1.1 (0.5)
Range	0.4–2.2
eGFR, ml/min per 1.73 m^2^	
Mean (SD)	74.0 (21.0)
Range	42–122
Serum creatinine, μmol/l	
Mean (SD)	99.8 (26.3)
Range	58–149
IgA,^a^ g/l	
Mean (SD)	3.1 (1.0)
Range	1.4–5.0
Gd-IgA1, mg/l	
Mean (SD)	6.6 (3.4)
Range	1.5–14.9
IgG,^b^ g/l	
Mean (SD)	10.7 (2.1)
Range	7.9–14.1
IgM,^c^ g/l	
Mean (SD)	1.1 (0.7)
Range	0.5–3.1
Hematuria, *n* (%)	13 (76.5%)

ACE, angiotensin-converting enzyme; ARB, angiotensin receptor blocker; BMI, body mass index; eGFR, estimated glomerular filtration rate; Gd-IgA1, galactose-deficient IgA1; SGLT2, sodium-glucose transporter-2; UACR, urine albumin-to-creatinine ratio; UPCR, urine protein-to-creatinine ratio; UPE, urine protein excretion.

a Normal range for IgA: 0.7–4.0 g/l.^10^

b Normal range for IgG: 7.0–16.0 g/l.^10^

c Normal range for IgM: 0.4–2.3 g/l.^10^

### Safety

At week 96, 16 of 17 (94.1%) participants had any AE and 11 of 17 (64.7%) had a treatment-related AE (Table 2). The time-adjusted incidence rate of AEs was 804.7 per 100 patient-years and 620.1 per 100 patient-years for the first 48 weeks and 96 weeks, respectively. The majority of AEs were mild or moderate, with only 3 of 17 (17.6%) participants having a grade ≥ 3 AE by week 96. One grade ≥ 3 AE, decreased white blood cell count, was considered related to mezagitamab by the investigator. The participant experienced a leukocyte count of 1.95 x 10^9^/l and neutrophil count of 1.07 x 10^9^/l on study day 22. Mezagitamab dosing was held until leukocytes and neutrophils recovered to baseline levels, which occurred by day 36, with no infection-related AEs during that period. Two participants experienced a grade ≥ 3 AE that was considered not related to mezagitamab—benign right breast mass and increased blood creatinine phosphokinase level. No serious AEs occurred, and no participants discontinued the study owing to an AE. The most common AEs occurring in ≥ 15% of participants were upper respiratory tract infection (8/17; 47.1%), COVID-19 (4/17; 23.5%), nasopharyngitis (4/17; 23.5%), oropharyngeal pain (4/17; 23.5%), and pyrexia (4/17; 23.5%).

**Table 2:** Summary of adverse events in the main study and LTE (*N* = 17)

Event	Number of participants experiencing event, *n* (%)
Any AE	16 (94.1)
AE related to study drug	11 (64.7)
Grade ≥ 3 AE	3 (17.6)
Grade ≥ 3 AE related to study drug	1 (5.9)
Serious AE	0
Treatment discontinuations because of AE	0
AEs related to infections	14 (82.4)
AEs occurring in ≥ 15% of participants	
Upper respiratory tract infection	8 (47.1)
COVID-19	4 (23.5)
Nasopharyngitis	4 (23.5)
Oropharyngeal pain	4 (23.5)
Pyrexia	4 (23.5)
Back pain	3 (17.6)
Cytokine release syndrome	3 (17.6)
Headache	3 (17.6)
Injection-site erythema	3 (17.6)

AE, treatment-emergent adverse event; LTE, long-term extension.

There were no serious infections or opportunistic infections reported, and no participants had their IgG serum level decrease below 350 mg/dl. There were no grade ≥ 3 or serious hypersensitivity events, nor anaphylaxis events.

Injection-related reactions were observed, typically with the first dose of mezagitamab, which were generally mild and self-limiting fever with or without constitutional symptoms. None of the injection-related reactions was serious or led to discontinuation of mezagitamab. Three non-serious, grade 1 AEs were reported with the term of “cytokine release syndrome.” All 3 events were limited to fever and/or constitutional symptoms with the first dose of mezagitamab that resolved either spontaneously or with acetaminophen/paracetamol. No hypotension or hypoxia were observed. Cytokine levels were not specifically sampled during these events. In the absence of hypotension, hypoxia, or biological evidence of cytokine release syndrome, these 3 AEs appear to be more consistent with injection-related reactions.

### Efficacy

A rapid reduction in proteinuria (as measured by a UPCR from a 24-hour urine collection and reported as a model-based geometric mean) was observed within a few weeks, with a percent change from baseline of −54.3 (95% CI, −65.7 to −39.1) at week 48 (Figure 3a). The reduction in proteinuria persisted through week 96, approximately 1.5 years after the last dose of mezagitamab, with a percent change from baseline of −53.4 (95% CI, −72.6 to −20.7) for the participants in the LTE observation group. Consistent with this observation, the UACR was also persistently reduced with a percent change from baseline of −60.2 (95% CI, −72.1 to −43.2) at week 48 and of −66.0 (−79.5 to −43.5) at week 96 (Figure 3b). Kidney function, as measured by eGFR, remained stable during the study (Figure 3c). The model-based mean (95% CI) change from baseline in eGFR was +1.9 (−2.0 to +5.8) at week 48 and +3.2 (−1.1 to +7.5) at week 96. Since all participants did not contribute data at the week 96 time point, to explore how robust the analysis was with different imputation strategies for missing data, a series of sensitivity analyses were performed. These analyses showed results generally consistent with the observed stabilization of eGFR at week 96 in the primary analysis (Supplementary Table S2). Following retreatment, the 2 retreated participants reached or exceeded the maximum reduction in UPCR that had been observed in year 1 (following the first cycle of treatment).

**Figure 3:**
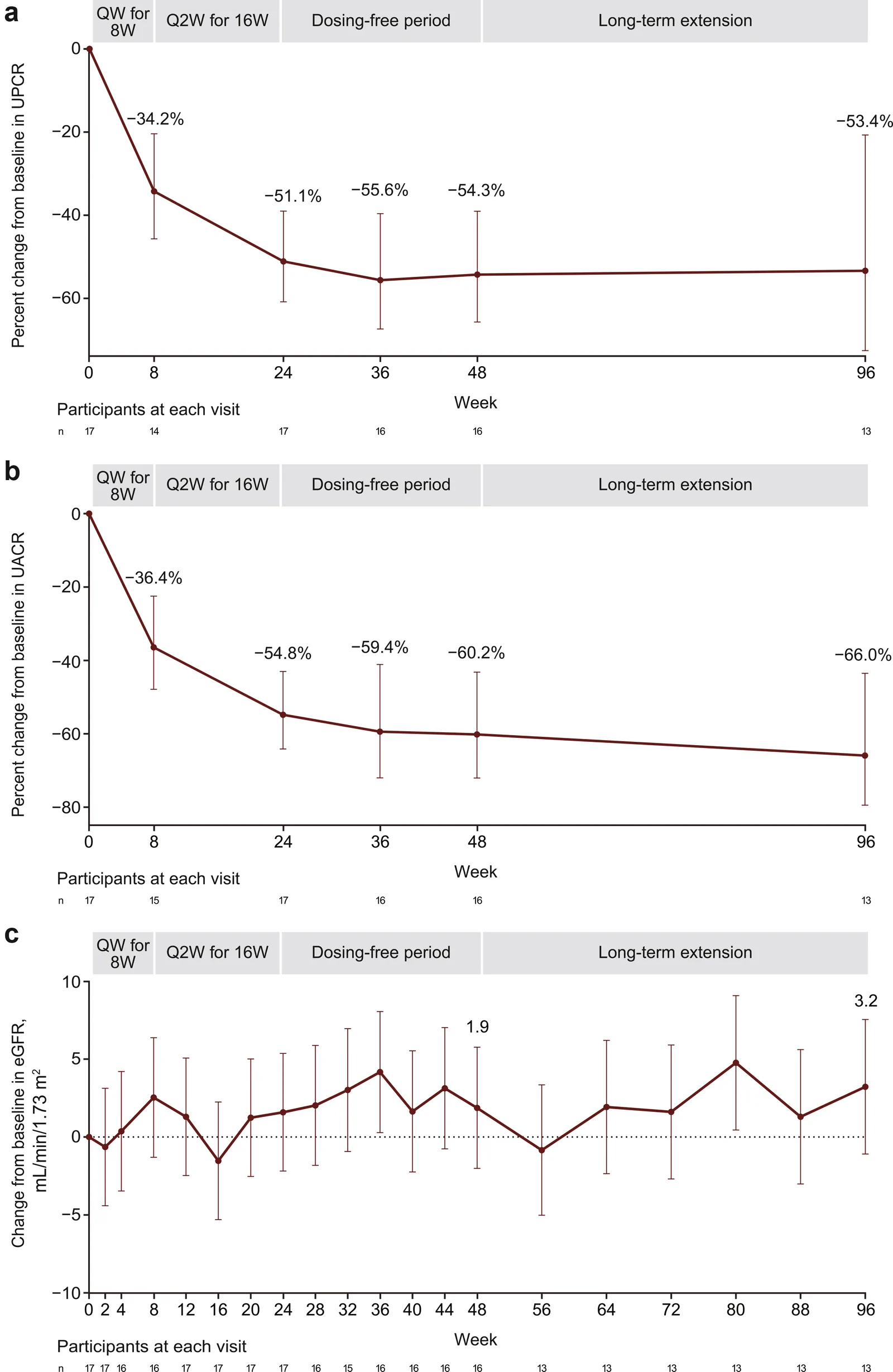
Change from baseline in (a) urine protein-to-creatinine ratio (UPCR), (b) urine albumin-to-creatinine ratio (UACR), and (c) estimated glomerular filtration rate (eGFR) in the main study and long-term extension (LTE) observation group. Mean and 95% CIs are shown. Panels a, b, c: mean was derived from a mixed-effects model for repeated measures over all postbaseline visits, with visit as a factor, and adjusted by baseline value and baseline-by-visit interaction. During the main study, mezagitamab was administered weekly and then biweekly, with the last dose at week (W) 22. The LTE observation period started after the week 48 visit in the main study and ended at week 96. QW, every week; Q2W, every 2 weeks.

Thirteen participants had hematuria at baseline (4 male; 9 female), defined as non-negative, nontrace blood. There was a resolution of hematuria in 5 of these 13 participants (39%; 95% CI, 12–65) at weeks 24 and 36, in 9 of 13 participants (69%; 95% CI, 44–94) at week 48, and in 6 of 10 participants (60%; 95% CI, 30–90) at week 96.

### Pharmacokinetics, Pharmacodynamics, and Immunogenicity

Mezagitamab serum concentrations increased steadily with each weekly dosing through week 8 and decreased from week 8 through week 24 during biweekly dosing (Supplementary Figure S1). The mean (SD) mezagitamab serum concentrations were 219.8 (65.7) μg/ml at week 8 and 123.1 (56.3) μg/ml at week 24. During the safety follow-up period, 16 of 17 (94.1%) participants had mezagitamab concentrations below the lowest limit of quantification (10 ng/ml) by week 48.

Four of 17 (23.5%) participants had treatment-emergent antidrug antibody at 1 or more visits through week 96. Post-treatment antidrug antibody did not appear to affect the PK of mezagitamab.

Rapid reductions were observed within days in serum IgA (Figure 4a), IgG (Figure 4b), and IgM (Figure 4c). At week 24, the mean (95% CI) percent change from baseline was −70.2 (−75.0 to −65.3) for IgA, −34.9 (−41.7 to −28.0) for IgG, and −39.3 (−47.4 to −30.6) for IgM. By week 48, Ig levels had begun to return toward baseline levels, with mean (95% CI) percent change from baseline for IgA of −52.4 (−58.2 to −46.2), for IgG of −18.9 (−26.7 to −11.3), and for IgM of −19.9 (−27.6 to −11.6). At week 96, in the long-term observation period, IgA levels continued to return toward baseline with a mean (95% CI) percent change from baseline of −28.4 (−33.8 to −23.1), whereas IgG and IgM levels had recovered baseline levels: 2.4 (−4.7 to 9.2) for serum IgG and 4.7 (−5.0 to 14.8) for serum IgM.

**Figure 4:**
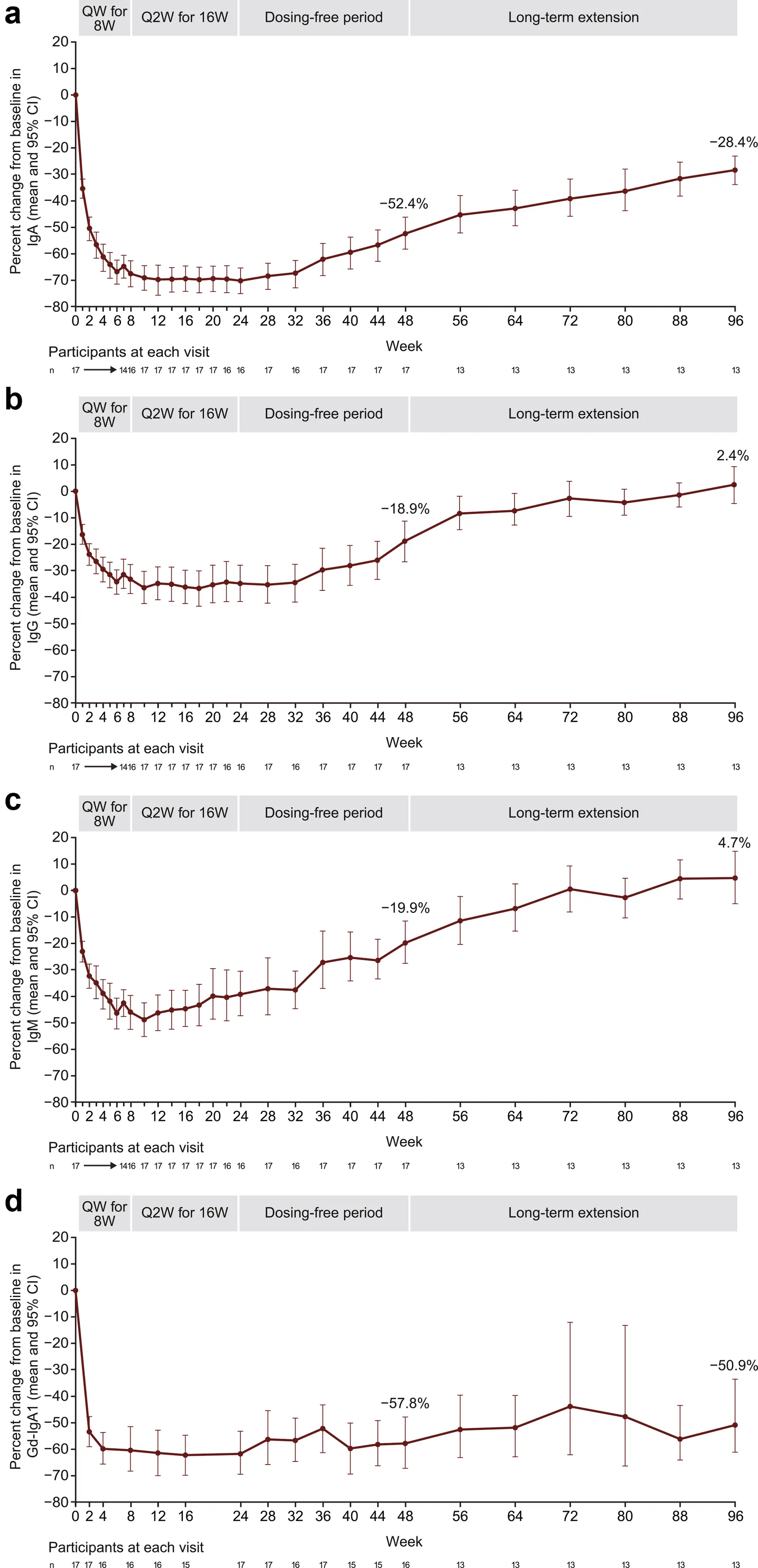
Change from baseline in (a) serum IgA, (b) serum IgG, (c) serum IgM, and (d) serum galactose-deficient IgA1 (Gd-IgA1) in the main study and long-term extension (LTE) observation group. Mean and 95% CIs are shown. During the main study, mezagitamab was administered weekly and then biweekly through week (W) 22. The LTE observation period started after the week 48 visit in the main study and ended at week 96. QW, every week; Q2W, every 2 weeks.

A rapid reduction in Gd-IgA1 was observed within days. At week 24, the mean (95% CI) percent change from baseline for Gd-IgA1 was −61.7 (−69.4 to −53.2), reaching nadir at approximately week 24 as well. This reduction was sustained through week 96 (Figure 4d), with a mean (95% CI) percent change from baseline of −57.8 (−67.2 to −47.8) at week 48 and −50.9 (−61.1 to −33.5) at week 96.

Reductions in IgA and IgG levels in the retreatment phase for the 2 retreated participants were comparable to those observed in the first treatment cycle, whereas reductions in Gd-IgA1 levels for these 2 participants reached or numerically exceeded the maximum reduction observed in the first cycle of treatment.

Flow cytometry data showed a rapid reduction in natural killer cells within days after treatment with mezagitamab that was sustained through week 24, with partial recovery toward baseline thereafter (Supplementary Figure S2). Leukocyte, neutrophil, and lymphocyte counts showed initial decreases, but returned to baseline during the dosing period (Supplementary Figure S3). Among lymphocyte subsets analyzed by flow cytometry, B cells exhibited a slight reduction (mean, ≤ 13%) with recovery after week 12, and T-cell subsets showed an increase above baseline from week 8 (CD8^+^ T cells) or week 12 (CD4^+^ T cells) through week 48 (Supplementary Table S3).

## Discussion

In this phase 1b study, mezagitamab was generally well tolerated as an add-on to standard-of-care therapy in participants with primary IgAN, with no serious AEs observed. Rapid decreases in proteinuria and Gd-IgA1 were accompanied by stable kidney function (eGFR) that was sustained 1.5 years after the last dose of mezagitamab, indicating a durable response.

Mezagitamab was administered via subcutaneous injection, and no serious injection-related reactions were observed. Injection-related reactions generally occurred with the first dose and were mostly events of grade 1 fever with or without constitutional symptoms; none led to drug discontinuation. Although Ig levels were reduced by mezagitamab treatment, IgG reductions were moderate (with a maximum reduction from baseline of approximately 37%; Figure 4b) and had largely recovered by the end of the study. Exploratory flow cytometry data suggested that overall levels of circulating B cells were largely unaffected, with a minimal and transient decrease, whereas T cells were expanded by treatment. Concordant with these observations, no opportunistic infections or serious infections were observed.

The recently updated, standard-of-care International Kidney Disease: Improving Global Outcomes Clinical Practice Guidelines focus on preventing the formation of IgA immune complexes with budesonide (Nefecon), systemic glucocorticoids, or a proliferation-inducing ligand and/or B-cell activating factor inhibitors, on complement inhibition, and managing IgAN-induced nephron loss with renin-angiotensin system inhibition or sparsentan and sodium glucose cotransporter 2 inhibitor.^5^, ^11^ However, current therapies for IgAN continue to leave an unmet need for less burdensome, subcutaneous therapies that allow patients with IgAN to have long-term preservation of kidney function with a substantial period of time off-treatment. Although a targeted-release formulation of budesonide has demonstrated that kidney function is generally maintained at baseline levels with 9 months of dosing, progressive loss of kidney function continues after the end of treatment.^12^ This suggests that additional therapies are needed to directly target mechanisms that sustain the disease.^13^ In this regard, the subset of antibody-producing cells that secrete the pathogenic Gd-IgA1 appear to be enriched in cells that express CD38,^14^, ^15^ including CD20^–^ long-lived plasma cells.^16^

The results reported here support the hypothesis that mezagitamab treatment can act to address multiple upstream and downstream mechanisms of IgAN disease pathophysiology, including potential reduction of Gd-IgA1, antibodies against Gd-IgA1, and effector cells. Mezagitamab treatment appears particularly tailored for IgA-mediated disease, as depletion of IgA was substantially stronger than IgG, which could be explained by IgA- and IgG-secreting plasma cells having distinct patterns of tissue localization and associated microenvironments that differentially impact their survival following binding of anti-CD38 antibodies.^17^ Furthermore, although overall IgA and IgG levels recovered toward baseline levels after the end of dosing, Gd-IgA1 remained stably depleted approximately 1.5 years after the end of dosing. This observation suggests the intriguing possibility that the sustained off-treatment effect of mezagitamab may be because of depletion of both short- and long-lived plasma cells responsible for continued secretion of the pathogenic Gd-IgA1.

Finally, we observed a rapid reduction in natural killer cells, which also express CD38. As natural killer cells may be involved in the pathogenesis of IgAN through cytotoxic effects on glomerular endothelial cells,^18^, ^19^ depletion of this cell type may also contribute to the rapid treatment response observed with mezagitamab.

Overall, the sustained depletion of Gd-IgA1, reduction of proteinuria, and stabilization of eGFR up to 72 weeks after the end of therapy with mezagitamab as an add-on to standard-of-care suggest a promising potential for a disease-modifying effect that was observed despite a varying background of standard therapies, including 6 participants taking an sodium-glucose cotransporter 2 inhibitor. A sustained reduction in UPCR was recently reported with the anti-CD38 antibody felzartamab administered i.v. in a phase 2a study in patients with IgAN.^20^ However, with mezagitamab, we observed that while IgA levels began to recover after the end of treatment, the reduction in Gd-IgA1 levels appeared sustained through 96 weeks, a pattern that has not been previously reported. It remains to be determined whether these differential treatment effects on IgA and Gd-IgA1 levels reflect differences in antibody affinity, epitope specificity, effector functions, and/or dosing between mezagitamab and felzartamab.

Strengths of this trial include its multicenter, global design, and the assessment of safety and efficacy 18 months after the last dose. However, the interpretation of the results is limited by the lack of a comparator group and the limited sample size that was not powered to test an efficacy hypothesis. Although this trial did not have a placebo-treated group, several previous clinical trials have consistently shown that kidney function continues to decline without treatment in a comparable population of patients with IgAN who are at risk for progression.^21^, ^22^, ^23^, ^24^ Although the trial results are promising, they should be confirmed in an appropriately powered, global, randomized clinical trial, which is in progress.

In conclusion, mezagitamab in combination with stable background therapy was generally well tolerated in this proof-of-concept study in participants with primary IgAN. Treatment resulted in sustained mean reductions in proteinuria and Gd-IgA1 levels, and stabilization of kidney function that persisted 1.5 years after the end of mezagitamab dosing. Further investigation of mezagitamab for IgAN is warranted and a confirmatory, pivotal stage trial is ongoing (NCT06963827). A subcutaneous therapy that allows long-term stabilization of kidney function off-treatment could provide a unique treatment option for patients with IgAN.

## Disclosure

JB has received consulting and speaker fees from Alnylam, argenx, Astellas Pharma, BioCryst, Calliditas Therapeutics, Chinook Therapeutics, Dimerix, Galapagos, Novartis, Omeros, Takeda, Travere, Vera Therapeutics, and Visterra; grant support from argenx, Calliditas Therapeutics, Chinook Therapeutics, Galapagos, GSK, Novartis, Omeros, Travere, and Visterra; support for clinical trials from Calliditas Therapeutics, Chinook Therapeutics, Novartis, Omeros, Vera Therapeutics, and Visterra; and support for research projects from argenx, Calliditas Therapeutics, Chinook Therapeutics, Galapagos, GSK, Novartis, Omeros, Travere, and Visterra.

DKK has received support for clinical trials from Chinook Therapeutics, Novartis, and Vera Therapeutics.

IP has received research funding from Takeda.

JR has received speaker fees from Alexion, AstraZeneca, CSL Seqirus, and Novartis. YSa has received honoraria from Kyowa Kirin, Novartis, and Otsuka.

MS has received honoraria from AstraZeneca, Boehringer Ingelheim, Kaneka, Kyowa Kirin, and Vantive.

YSu has received research funding from Aurinia Pharmaceuticals, Japan Kidney Association, Mebix, Novartis, Pfizer, Rona Bioscience, Takeda, Tokiwa, and Travere; consulting fees from Alexion, Alpine Immune Sciences, argenx, BioCryst, Chinook Therapeutics, George Clinical, Novartis, Otsuka, and Renalys Pharma; and payment or honoraria from AstraZeneca, Boehringer Ingelheim, Chinook Therapeutics, Daiichi Sankyo, Kyowa Kirin, Mitsubishi Tanabe Pharma, Novartis, and Otsuka.

ID, MKF, CL, VAN, and PP are employees of Takeda Development Center Americas, Inc., and stockholders of Takeda Pharmaceutical Company Limited.

## Supplementary Material

Supplementary file (PDF) **Figure S1.** Mezagitamab serum concentrations over time in the main study. **Figure S2.** Change from baseline in natural killer (NK) cells over time in the main study and long-term extension observation group. **Figure S3.** (a) Leukocyte count, (b) neutrophil count, and (c) lymphocyte count over time in the main study and long-term extension observation group. **Table S1.** Oxford classification scores at baseline for study participants, **Table S2.** Sensitivity analyses for mean change from baseline in estimated glomerular filtration rate (eGFR) at week 96. **Table S3.** Mean (SD) percent change from baseline in B cells and T cells. CONSORT checklist

## Footnotes

Supplementary file (PDF)
**Figure S1.** Mezagitamab serum concentrations over time in the main study. **Figure S2.** Change from baseline in natural killer (NK) cells over time in the main study and long-term extension observation group. **Figure S3.** (a) Leukocyte count, (b) neutrophil count, and (c) lymphocyte count over time in the main study and long-term extension observation group. **Table S1.** Oxford classification scores at baseline for study participants. **Table S2.** Sensitivity analyses for mean change from baseline in estimated glomerular filtration rate (eGFR) at week 96. **Table S3.** Mean (SD) percent change from baseline in B cells and T cells. CONSORT checklist.
