# Supplementary material for: Clinical Trial of Open-Label Mezagitamab in IgAN: Final 96-Week Results
Source: Kidney Int Rep. 2026 Oct 7;12(1):107128. doi: 10.1016/j.ekir.2026.107128 (PMC13761348; doi:10.1016/j.ekir.2026.107128)
Supplement: Supplementary file 1 [file mmc1.pdf]

SUPPLEMENTARY MATERIAL

SUPPLEMENTARY TABLES

Supplementary Table S1. Oxford classification scores at baseline for study participants

**Oxford classification score available**	**n (%)**
Yes	15 (88.2)
No	2 (11.8)
**Mesangial hypercellularity score**	
M0: <50% of glomeruli showing mesangial hypercellularity	7 (41.2)
M1: >50% of glomeruli showing mesangial hypercellularity	8 (47.1)
Missing	2 (11.8)
**Endocapillary hypercellularity score**	
E0: No endocapillary hypercellularity	7 (41.2)
E1: Any glomeruli showing endocapillary hypercellularity	8 (47.1)
Missing	2 (11.8)
**Segmental glomerulosclerosis score**	
S0: Absent	5 (29.4)
S1: Present In any glomeruli	10 (58.8)
Missing	2 (11.8)
**Tubular atrophy/interstitial fibrosis score**	
T0: 0–25% of cortical area	14 (82.4)
T1: 26–50% of cortical area	1 (5.9)
Missing	2 (11.8)
**Cellular or fibrocellular crescents score**	
C0: Absent	8 (47.1)
C1: 0–25% of glomeruli	5 (29.4)
Missing	4 (23.5)

Supplementary Table S2. Sensitivity analyses for mean change from baseline in estimated glomerular filtration rate (eGFR) at week 96

	**Participant 1**	**Participant 2**	**Participant 3**
Intercurrent event (ICE)	Used rescue therapy and did not enter LTE due to lack of favorable proteinuric respose	Did not enter LTE due to lack of favorable proteinuric response	Retreated at week 48
ICE study visit	Week 28	Week 48	Week 48
Data availability after ICE	Data missing beyond week 48	Data missing beyond week 48	No missing data
**eGFR change from baseline at week 96** (mean and 95% CI, mL/min/1.73 m^2)			
Primary analysis<br>+3.22 (−1.09, +7.54)	Data after ICE set to missing and model implicitly imputed missing data under MAR	Model implicitly imputed missing data under MAR after ICE	Data after ICE set to missing and model implicitly imputed under MAR
Sensitivity analysis 1: retreatment data included<br>+3.73 (−0.47, +7.92)	Same as above	Same as above	Observed data used as if ICE did not occur
Sensitivity analysis 2: retreatment data included + conservative assumptions for non-responders<br>+2.23 (−1.76, +6.21)	Imputed missing data by a constant rate of decline at 4 units/year after week 48*	Imputed missing data by a constant rate of decline at 4 units/year after ICE	Observed data used as if ICE did not occur
Sensitivity analysis 3: conservative assumptions for non-responders and for the retreated participant<br>+1.41 (−2.50, +5.32)	Same as above	Same as above	Data after ICE set to missing and imputed by a constant rate of decline at 4 units/year

Data are presented as mean (95% CI). *For participant 1, the observed data between the use of prohibited therapy and the week-48 visit declined faster than the imputation assumption, as was conservatively retained for analysis, with missing data after the week-48 visit imputed as indicated. For the participant who did not enter the LTE due to safety reasons, data missing beyond week 48 were imputed under MAR after ICE in the primary analysis and in the sensitivity analyses. CI, confidence interval; LTE, long-term extension; MAR, missing at random.

Supplementary Table S3. Mean (SD) percent change from baseline in B cells and T cells

Time after first dose, weeks	n	Mean % (SD)
		**CD3- CD19+ B cells**
2	17	−8.1 (24.34)
4	16	−12.8 (27.46)
8	16	−13.0 (21.90)
12	17	−2.6 (21.05)
16	16	3.3 (25.48)
24	17	9.5 (36.44)
36	17	19.3 (35.34)
48	17	16.3 (25.87)

Time after first dose, weeks		Mean % (SD)		
	**n**	**CD3+ T cells**	**CD3+ CD4+ T cells**	**CD3+ CD8+ T cells**
2	17	−6.9 (17.70)	−2.5 (18.61)	−10.1 (20.38)
4	16	−3.5 (24.56)	−2.1 (26.55)	−2.7 (24.56)
8	16	21.2 (49.56)	6.0 (45.64)	48.0 (68.35)
12	17	42.9 (53.67)	18.2 (49.15)	88.2 (91.35)
16	16	40.3 (45.83)	15.6 (35.14)	84.4 (87.43)
24	17	40.9 (33.90)	19.1 (32.92)	80.5 (59.88)
36	17	56.3 (37.88)	22.5 (26.34)	114.7 (83.51)
48	17	48.6 (40.77)	14.4 (23.24)	107.0 (99.23)

SD, standard deviation.

SUPPLEMENTARY FIGURES

Supplementary Figure S1. Mezagitamab serum concentrations over time in the main study.

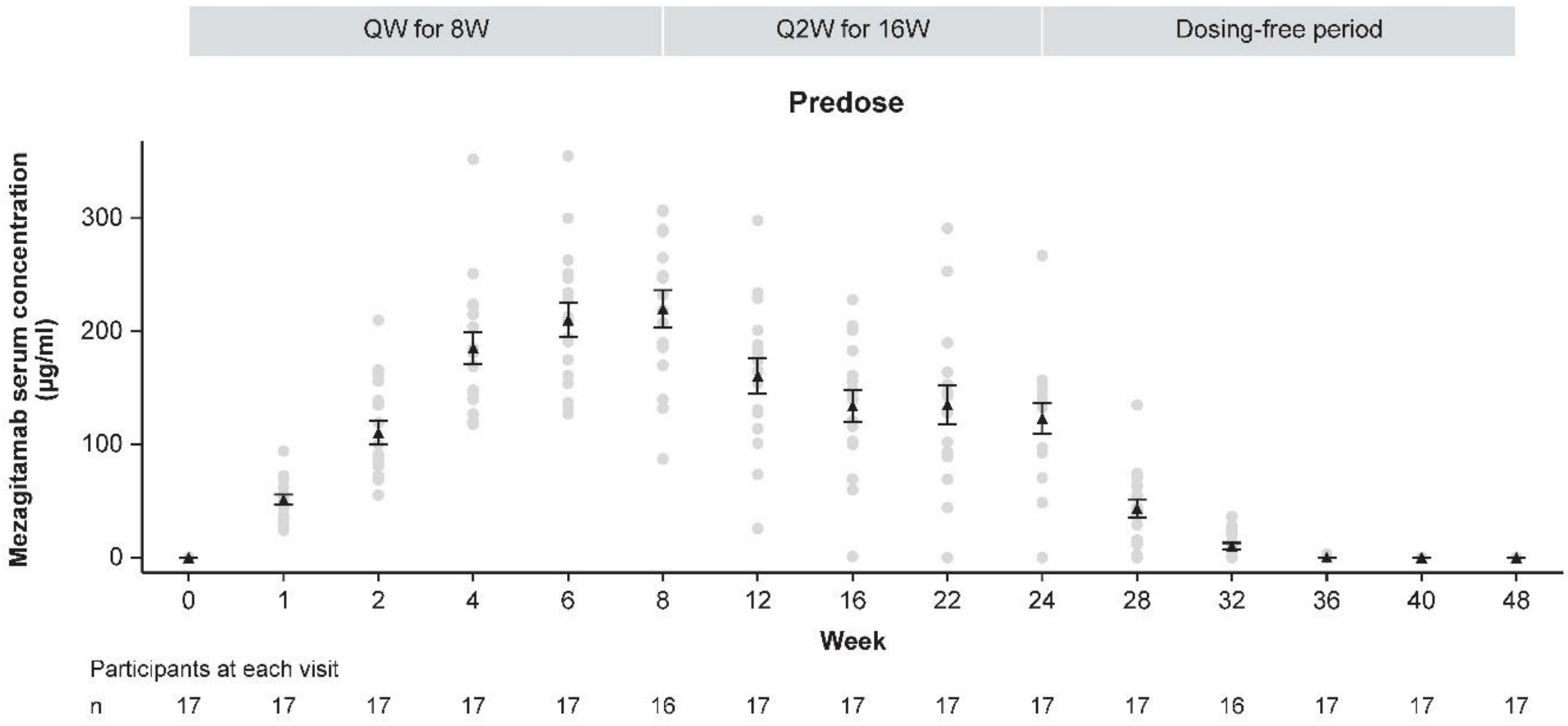

Data are shown as mean and SE with individual results as gray dots for participants with measurable mezagitamab concentration. Concentration values below the lower limit of quantification (10 ng/ml) were assigned a value of zero for calculation of summary statistics. One participant had a mezagitamab concentration of 10.4 ng/ml at week 48. QW, every week; Q2W, every 2 weeks; SE, standard error.

Supplementary Figure S2. Change from baseline in natural killer (NK) cells over time in the main study.

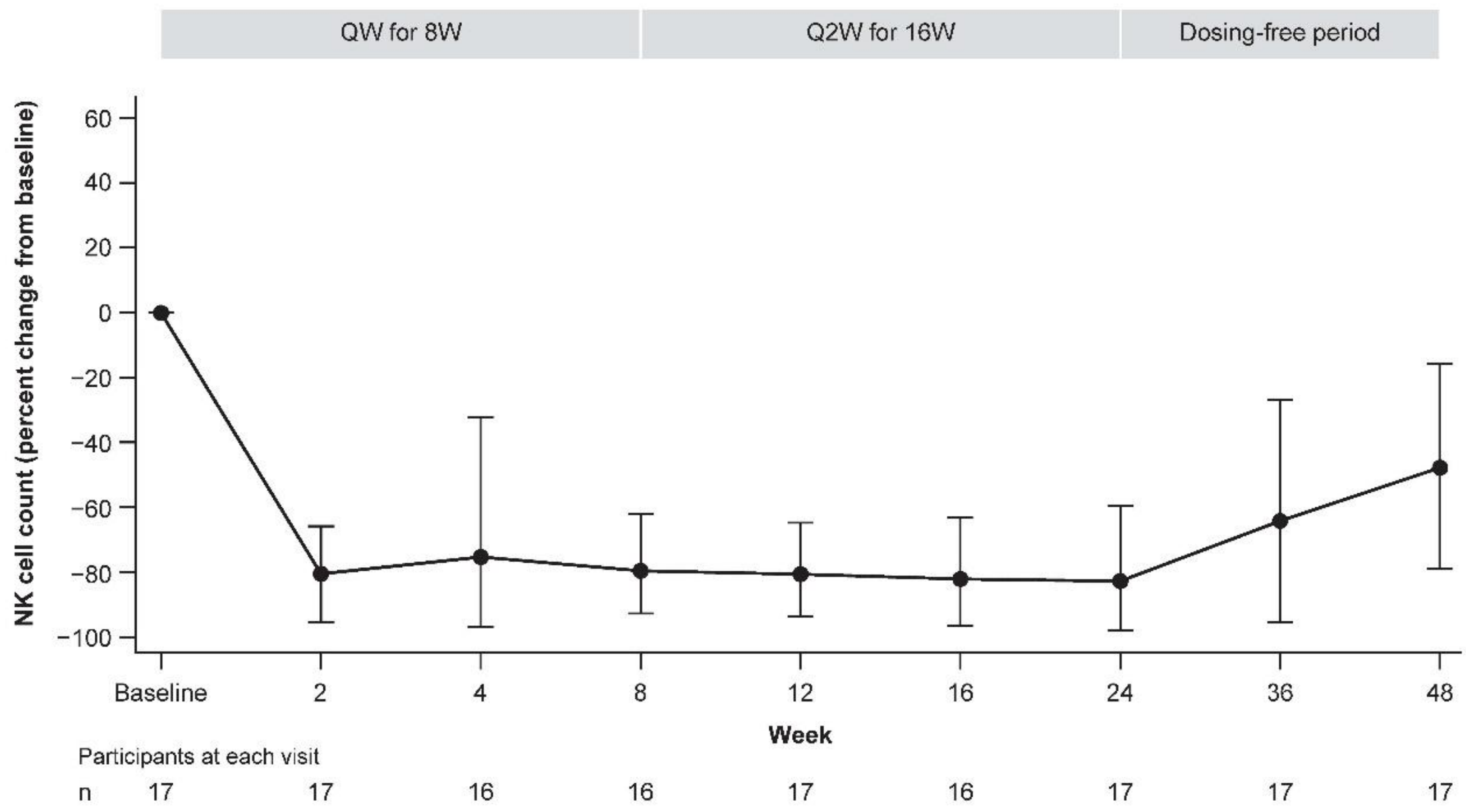

Circulating C3- CD56+ CD16+ NK cells were measured over time by flow cytometry. Black dots represent the mean and error bars represent 95% confidence intervals. QW, every week; Q2W, every 2 weeks.

Supplementary Figure S3. (a) Leukocyte count, (b) neutrophil count, and (c) lymphocyte count over time in the main study and long-term extension observation group.

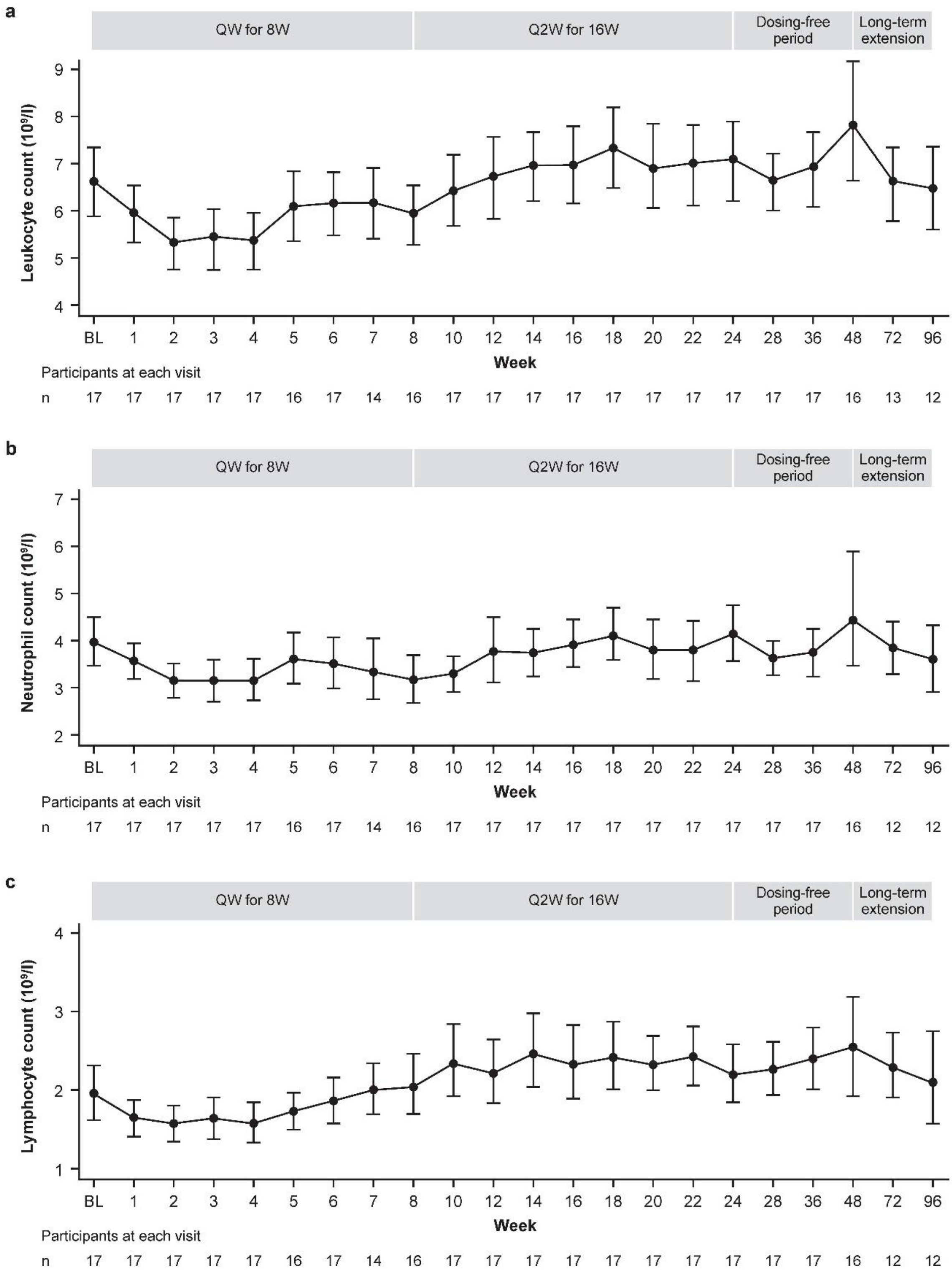

Dots and error bars represent mean and 95% confidence intervals. BL, baseline; QW, every week; Q2W, every 2 weeks.

Section/topic	No	CONSORT 2025 checklist item description	Reported on page no.
**Title and abstract**			
Title and structured abstract	1a	Identification as a randomized trial	N.A.
	1b	Structured summary of the trial design, methods, results, and conclusions	p. 3-4
**Open science**			
Trial registration	2	Name of trial registry, identifying number (with URL) and date of registration	p. 4
Protocol and statistical analysis plan	3	Where the trial protocol and statistical analysis plan can be accessed	p. 20-21
Data sharing	4	Where and how the individual de-identified participant data (including data dictionary), statistical code and any other materials can be accessed	p. 20-21
Funding and conflicts of interest	5a	Sources of funding and other support (e.g., supply of drugs), and role of funders in the design, conduct, analysis and reporting of the trial	p. 20
	5b	Financial and other conflicts of interest of the manuscript authors	p. 19-20
**Introduction**			
Background and rationale	6	Scientific background and rationale	p. 5
Objectives	7	Specific objectives related to benefits and harms	p. 5-6
**Methods**			
Patient and public involvement	8	Details of patient or public involvement in the design, conduct and reporting of the trial	N.A.
Trial design	9	Description of trial design including type of trial (e.g., parallel group, crossover), allocation ratio, and framework (e.g., superiority, equivalence, non-inferiority, exploratory)	p. 6-7
Changes to trial protocol	10	Important changes to the trial after it commenced including any outcomes or analyses that were not prespecified, with reason	N.A.
Trial setting	11	Settings (e.g., community, hospital) and locations (e.g., countries, sites) where the trial was conducted	p. 6
Eligibility criteria	12a	Eligibility criteria for participants	p. 6-8
	12b	If applicable, eligibility criteria for sites and for individuals delivering the interventions (e.g., surgeons, physiotherapists)	N.A.
Intervention and comparator	13	Intervention and comparator with sufficient details to allow replication. If relevant, where additional materials describing the intervention and comparator (e.g., intervention manual) can be accessed	p. 6-7
Outcomes	14	Prespecified primary and secondary outcomes, including the specific measurement variable (e.g., systolic blood pressure), analysis metric (e.g., change from baseline, final value, time to event), method of aggregation (e.g., median, proportion), and time point for each outcome	p. 8-9
Harms	15	How harms were defined and assessed (e.g., systematically, non-systematically)	p. 9
Sample size	16a	How sample size was determined, including all assumptions supporting the sample size calculation	p. 9
	16b	Explanation of any interim analyses and stopping guidelines	N.A.
Randomization:			
Sequence generation	17a	Who generated the random allocation sequence and the method used	N.A.

	17b	Type of randomization and details of any restriction (e.g., stratification, blocking and block size)	N.A.
Allocation concealment mechanism	18	Mechanism used to implement the random allocation sequence (e.g., central computer/telephone; sequentially numbered, opaque, sealed containers), describing any steps to conceal the sequence until interventions were assigned	N.A.
Implementation	19	Whether the personnel who enrolled and those who assigned participants to the interventions had access to the random allocation sequence	N.A.
Blinding	20a	Who was blinded after assignment to interventions (e.g., participants, care providers, outcome assessors, data analysts)	N.A.
	20b	If blinded, how blinding was achieved and description of the similarity of interventions	N.A.
Statistical methods	21a	Statistical methods used to compare groups for primary and secondary outcomes, including harms	p. 8-9
	21b	Definition of who is included in each analysis (e.g., all randomized participants), and in which group	p. 8-9
	21c	How missing data were handled in the analysis	p. 8-9
	21d	Methods for any additional analyses (e.g., subgroup and sensitivity analyses), distinguishing prespecified from post hoc	Suppl Table S2.
**Results**			
Participant flow, including flow diagram	22a	For each group, the numbers of participants who were randomly assigned, received intended intervention, and were analyzed for the primary outcome	p. 10, Figure 2
	22b	For each group, losses and exclusions after randomization, together with reasons	p. 10, Figure 2
Recruitment	23a	Dates defining the periods of recruitment and follow-up for outcomes of benefits and harms	N.A.
	23b	If relevant, why the trial ended or was stopped	N.A.
Intervention and comparator delivery	24a	Intervention and comparator as they were actually administered (e.g., where appropriate, who delivered the intervention/comparator, how participants adhered, whether they were delivered as intended (fidelity))	p. 10, Figures 1, 2, Table 2
	24b	Concomitant care received during the trial for each group	p. 6
Baseline data	25	A table showing baseline demographic and clinical characteristics for each group	Table 1, Table S1
Numbers analyzed, outcomes and estimation	26	For each primary and secondary outcome, by group:<br>● the number of participants included in the analysis<br>● the number of participants with available data at the outcome time point<br>● result for each group, and the estimated effect size and its precision (such as 95% confidence interval)<br>● for binary outcomes, presentation of both absolute and relative effect size	Table 2, Figures 3, 4, Figure S1 p. 10-13
Harms	27	All harms or unintended events in each group	Table 2, p. 10-11
Ancillary analyses	28	Any other analyses performed, including subgroup and sensitivity analyses, distinguishing pre-specified from post hoc	Suppl. Tables S2, S3; Figures S2, S3; p. 13-14
**Discussion**			
Interpretation	29	Interpretation consistent with results, balancing benefits and harms, and considering other relevant evidence	p. 15-18
Limitations	30	Trial limitations, addressing sources of potential bias, imprecision, generalizability, and, if relevant, multiplicity of analyses	p. 17

Citation: Hopewell S, Chan AW, Collins GS, Hróbjartsson A, Moher D, Schulz KF, et al. CONSORT 2025 Statement: updated guideline for reporting randomised trials. BMJ. 2025; 388:e081123.
https://dx.doi.org/10.1136/bmj-2024-081123
© 2025 Hopewell et al. This is an Open Access article distributed under the terms of the Creative Commons Attribution License (https://creativecommons.org/licenses/by/4.0/), which permits unrestricted use, distribution, and reproduction in any medium, provided the original work is properly cited.
